# Caliber-Persistent Artery

**DOI:** 10.1155/2015/747428

**Published:** 2015-09-06

**Authors:** Sabrina Araújo Pinho Costa, Marcelo Martinson Ruiz, Shajadi Pardo Kaba, Giovanna Piacenza Florezi, Celso Augusto Lemos Júnior, Andréa Lusvarghi Witzel

**Affiliations:** ^1^Department of Stomatology, School of Dentistry, University of São Paulo, Avenida Professor Lineu Prestes, 2227 Cidade Universitária, 05508-000 São Paulo, SP, Brazil; ^2^School of Dentistry, University of São Paulo, Avenida Professor Lineu Prestes, 2227 Cidade Universitária, 05508-000 São Paulo, SP, Brazil; ^3^Department of Stomatology Oral Medicine, School of Dentistry, University of São Paulo, Avenida Professor Lineu Prestes, 2227 Cidade Universitária, 05508-000 São Paulo, SP, Brazil; ^4^Foundation for Scientific and Technological Development of Dentistry, School of Dentistry, University of São Paulo, Avenida Professor Lineu Prestes, 2227 Cidade Universitária, 05508-000 São Paulo, SP, Brazil

## Abstract

Caliber-persistent artery (CPLA) of the lip is a common vascular anomaly in which a main arterial branch extends to the surface of the mucous tissue with no reduction in its diameter. It usually manifests as pulsatile papule, is easily misdiagnosed, and is observed more frequently among older people, suggesting that its development may involve a degenerative process associated with aging; CPLA is also characterized by the loss of tone of the adjacent supporting connective tissue. Although the diagnosis is clinical, high-resolution Doppler ultrasound is a useful noninvasive tool for evaluating the lesion. This report describes the case of a 58-year-old male patient who complained of a lesion of the lower lip with bleeding and recurrent ulceration. The patient was successfully treated in our hospital after a diagnosis of CPLA and is currently undergoing a clinical outpatient follow-up with no complaints.

## 1. Introduction

A caliber-persistent artery (CPLA) of the lip is a primary arterial branch that enters the submucosal tissue and shows no reduction in its diameter [1, 2]. This vascular anomaly has been described in the stomach and jejunum under different designations, including arterial malformation, Dieulafoy's disease, and cirsoid aneurysm, among others [3, 4].

This type of abnormal artery was first described in the oral cavity in 1973 by Howell and Freeman, who designated it as “prominent inferior labial artery” [5], and this study was followed by a series of other studies that used the term “caliber-persistent artery” [1, 2, 6–15].

The lesion presents as a papular, arcuate, or linear elevation with a normal or bluish pale color. In general, the artery becomes inconspicuous when the lip is stretched. The characteristic feature of this disorder is lateral and vertical pulsation. The lesion is initially asymptomatic and usually diagnosed during a routine clinical examination. The patient may occasionally notice an increase in the pulse volume in the lip [3].

Microscopically, the lesion is characterized by a normal muscular artery with an irregular lumen and a size that is unusual for its location in the submucosa [5, 12].

CPLAs occur almost exclusively on the lip mucosa and can affect both the upper and lower lips [1, 13, 14], and some patients can present with bilateral lesions or lesions in both lips [2, 12]. The average age of onset is approximately 50 years, and the disorder affects both genders in equal proportions.

Some cases may be associated with ulceration of the mucosal lining, and other rare cases may occur adjacent to a lip squamous cell carcinoma. CPLAs are occasionally associated with ulceration [6, 8, 9] and can be clinically diagnosed as squamous cell carcinoma [9]. Miko et al. [7] described CPAs as being associated with squamous cell carcinoma; however, it is unlikely that the cancer is associated with the CPLA. In addition, this association has not been found in other cases [1, 14]. A CPLA can be distinguished clinically from oral squamous cell carcinoma by the lack of fixation and hardening [12]. To explain the cause of ulceration, Howell and Freeman suggested that arterial pulse pressure causes ischemia in the submucosal tissue, which can result in the formation of ulcers [15]. Furthermore, the elevated nature of the swelling in CPLAs facilitates chronic trauma, which can lead to ulceration.

The factors that contribute to lip ulceration or erosion in CPLAs include accidental or chronic vascular atrophy or ectasia in the elderly, ischemia due to the increased pressure of a pulsating superficial submucosal artery, medications, chronic actinic cheilitis, and arteriosclerosis [12].

Although there have been reports of CPLAs in the stomach and other areas of the gastrointestinal tract, with some cases involving hemorrhage and death, no cases of bleeding in a CPLA of the lip have been reported. However, severe blood loss is possible due to trauma to the lip or excision without the identification of this tortuous artery. Therefore, recognition of the presence of this condition in the oral cavity is extremely important due to the risk of bleeding during oral and maxillofacial surgery [2].

Cases of CPLAs found in the lower and upper lips have involved dilatation of the inferior labial artery and superior labial artery, respectively [2, 13, 15].

## 2. Case Report

A 58-year-old male patient presented with swelling in the lower lip with bleeding and recurrent ulceration. The patient complained of swelling for two months before the diagnosis when he noticed the presence of a traumatic ulcer in the region. The swelling increased in size after it was first noticed and was accompanied by pain on palpation. The patient exhibited no systemic changes.

**Figure 1:**
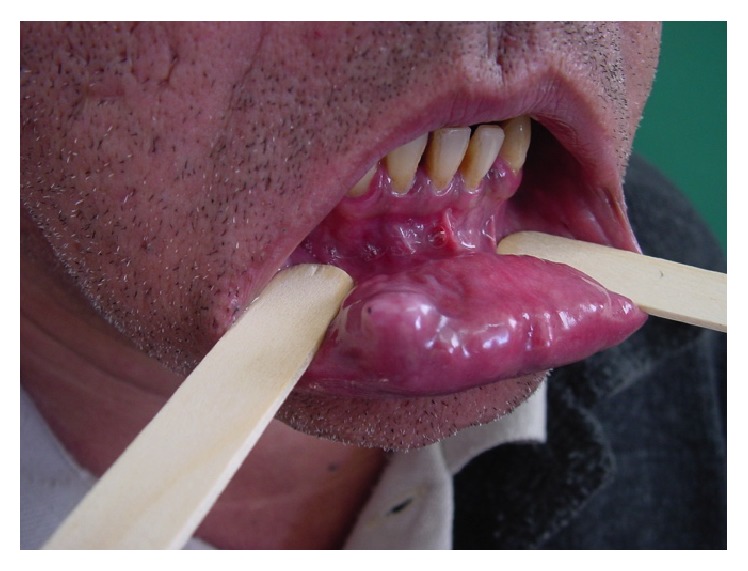
The bullous lesion of soft consistency characterized by the presence of a fistula in the mucosal surface and increased volume is shown.

**Figure 2:**
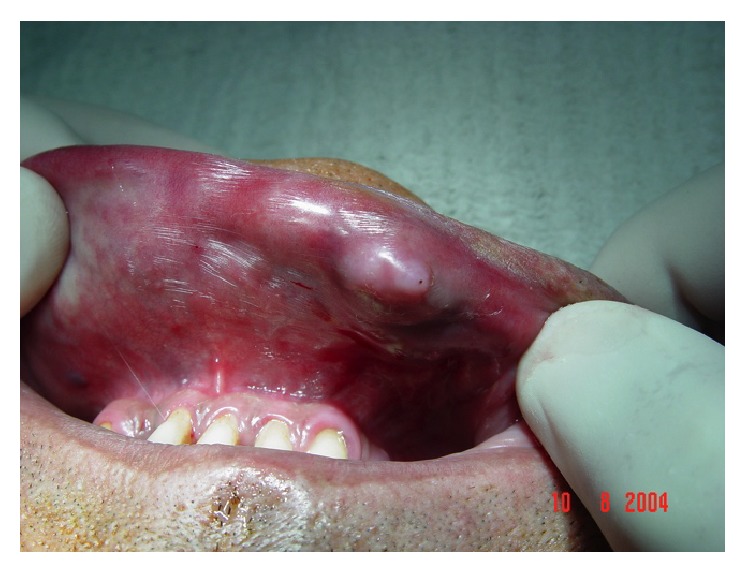
Peripheral progressive increase of the main bullous lesion.

A clinical examination indicated the presence of a bullous lesion along the left lower lip vermilion without well-defined margins and with the following characteristics: soft consistency, smooth surface, having same color as the mucosa, being approximately 1 cm at its largest diameter, and presence of a fistula (Figures 1 and 2). The lesion was not evident on radiographic examination because it was a soft-tissue lesion.

The differential diagnoses included mucocele, traumatic fibroma, canalicular adenoma, leiomyoma, and hemangioma.

The patient reported no past trauma or damaging oral habits; in addition, no clinical evidence suggestive of the presence of vascular proliferation in the adjacent tissue was found. Considering these data, the patient was clinically diagnosed with a CPLA of the lip.

**Figure 3:**
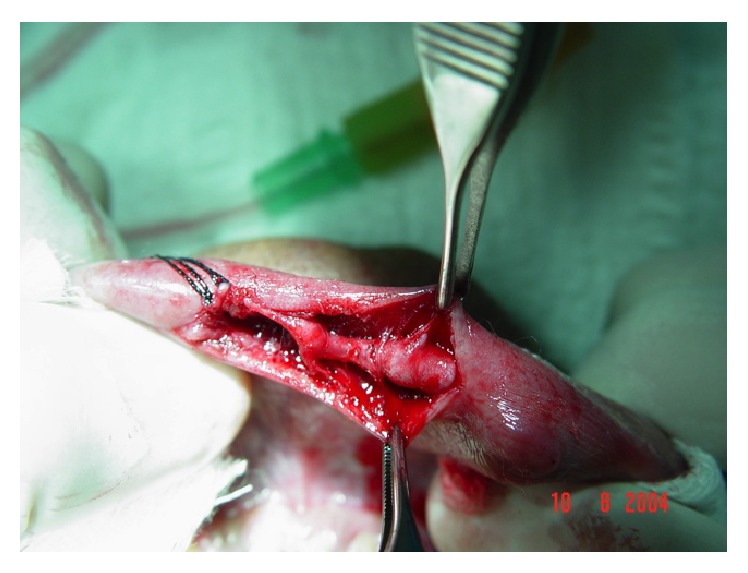
Procedure for excision of the superficial vascular branch, starting with the ligature of the main branches.

**Figure 4 fig4:**
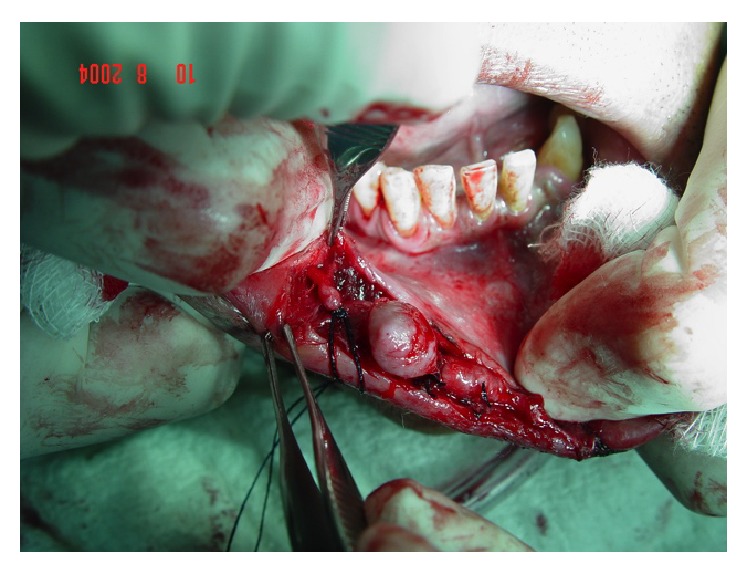
Ligature of the accessory vascular branches.

**Figure 5 fig5:**
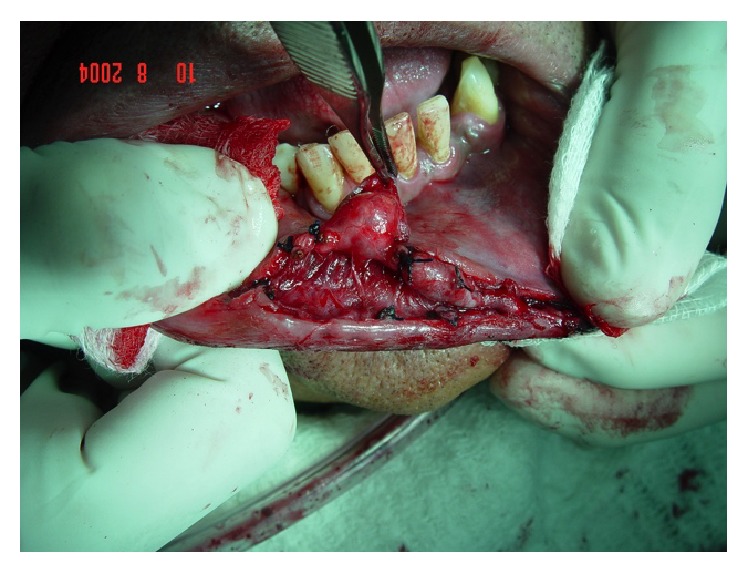
Isolation of the main vascular branch.

**Figure 6 fig6:**
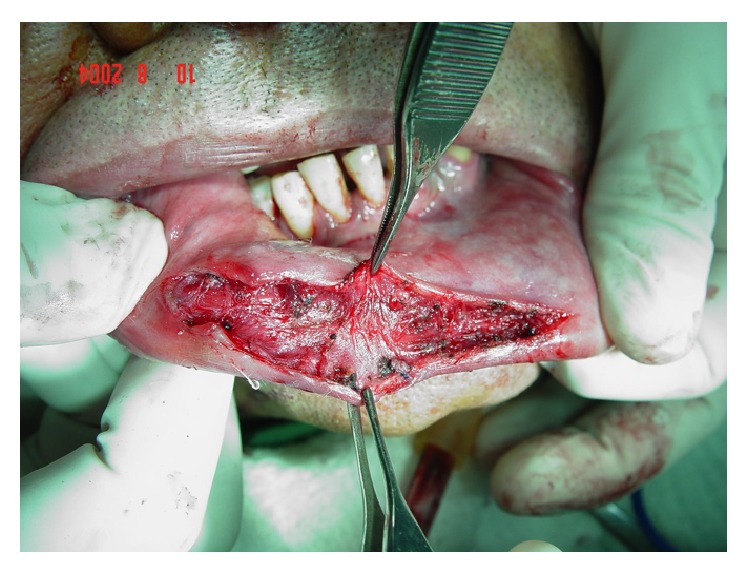
Surgical bed after excision of the artery revealing the absence of bleeding.

**Figure 7 fig7:**
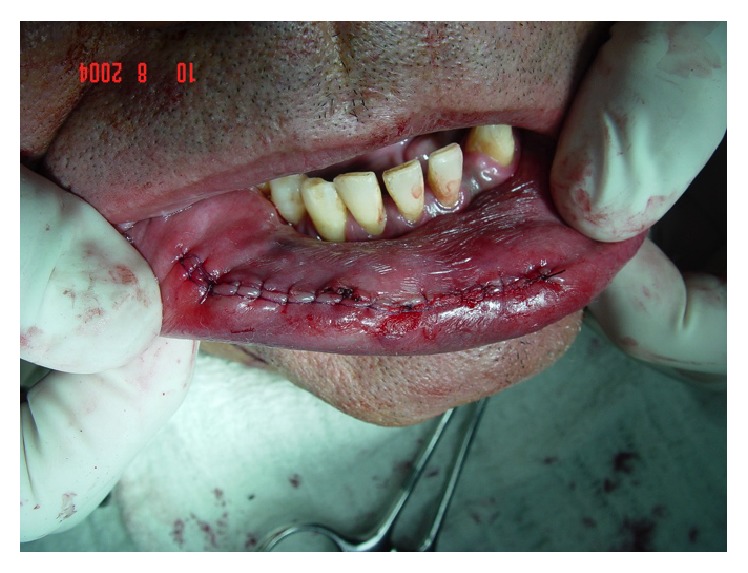
Continuous suture with resorbable thread.

**Figure 8:**
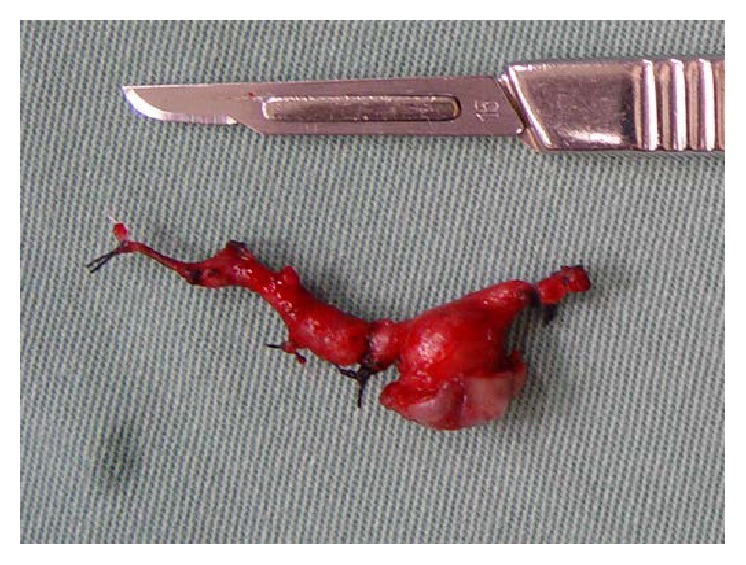
Specimen containing the ligature threads of the main branch.

The pulsating area was surgically explored under local anesthesia, and an approximately 1 mm CPA was detected in the submucosal layer. Subsequently, the artery was isolated using 2.0 cotton thread, and its most superficial portion was excised. Upon cleaning and irrigating the surgical cavity with 0.9% saline, the mucosa was sutured with 4.0 silk thread (Figures 3 [Fig fig4] [Fig fig5] [Fig fig6] [Fig fig7]–8). Antibiotic, anti-inflammatory, and analgesic drugs were prescribed in the postoperative period, during which there were no complications.

**Figure 9:**
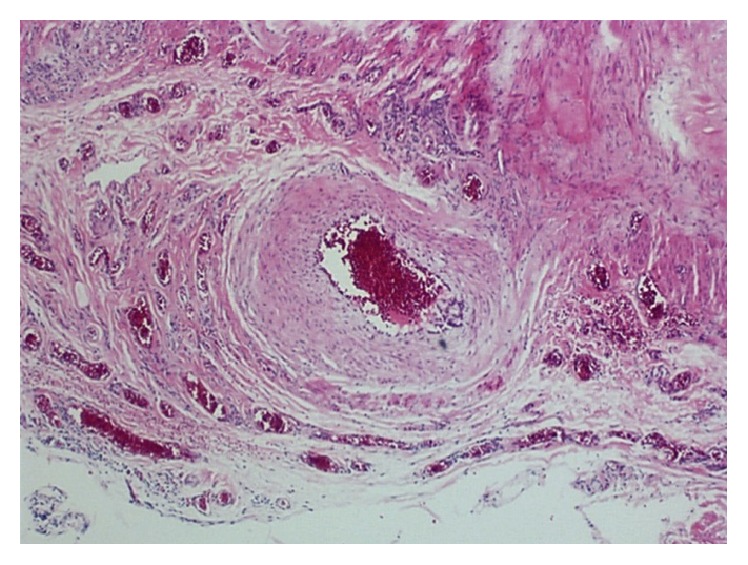
Microscopic examination of the specimen. No cellular changes were observed; only a normal cell structure of the arterial section was present (Hematoxylin-eosin, 40x).

The surgical specimen was sent for anatomopathological examination and identified as an arterial section, which, considering its unusually superficial position, provided a conclusive diagnosis of a CPLA in the lip mucosa (Figure 9).

## 3. Discussion

The descriptive term CPLA was initially used to describe a vascular anomaly in the stomach submucosa. The first studies on the subject indicated that the vascular anomaly in the mouth was associated with mucosal ulceration, possibly due to the ischemia caused by the vascular pulsation in the mucosa [6, 8], and this ulceration led to tissue necrosis and ulceration. Miko et al. [6] reported three cases of CPLA of the lower lip clinically diagnosed as ulcers that resembled squamous cell carcinoma. A few years later, these authors reported another case of CPLA [6] that was associated with squamous cell carcinoma of the lower lip. Considering these results, these authors suggested that the chronic ulcer produced by this condition stimulated a malignant epithelial transformation.

In 1985, Marshall and Leppard described a case of CPLA that also affected the lower lip and was associated with an ulcer with a progression period of five months. These authors performed surgery with safety margins because the ulcer was clinically compatible with squamous cell carcinoma; however, a histopathological examination indicated the presence of a benign lesion.

In that report, the clinical aspects included a pulsatile tubular lesion on palpation, and an ulcer was observed in the mucosa upon visual inspection, in contrast to the results of other studies that found no association with ulcers [5, 10, 12].

Although an angiographic examination was not performed, the case reported herein showed characteristics similar to those described by Howell, Freeman, and Jaspers [5, 9]: unilaterality, a location near the mucosal surface, and the presence of a nonulcerated node in the labial mucosa. Miko et al. [6] stated, “We have never found calibre persistent arteries under intact vermilion border, but always associated with chronic lip ulcers.”

Ulceration is thought to occur as a result of “pulsating pressure exerted by the artery on the epithelium” in the labial mucosa. Senile atrophy and/or solar damage have been listed as possible contributing factors. Solar damage is evident but not relevant to our case because the labial mucosa is usually exposed to this factor. The patient had no history of trauma but was a smoker. However, considering that the lesion was located in the occlusal plane, it was chronically exposed to occlusal trauma with recurrent bleeding. The patient became aware of his injury when the ulcers began to appear. A congenital CPLA malformation of the lip was likely but tended to remain unnoticed until mucosal ulceration began to occur. In previous reports, the youngest patient with a lip ulcer diagnosed as CPLA was 56 years old, and the average age was 73 years. Howell and Freeman [5] reported this disorder only in the 40–70-year-old group, which is consistent with the results of our study in that the patient described was 58 years old that is young compared to the group of patients with ulcers.

Clinically, the differential diagnosis of CPLA includes varicose veins and hemangioma when the lesion is visible, in addition to the presence of nonvascular entities, including mucocele and fibroma [2, 13]. A clinical diagnosis can be made when the lesion is visible by palpation or is pulsatile [13]. To confirm the diagnosis, most authors have used surgical biopsy, which carries a risk of bleeding [1, 5–9, 12, 14]. However, Lovas and Goodday [10] used angiography, and other authors advocate the use of noninvasive techniques, including Doppler ultrasound [2].

Different theories have been postulated to explain the etiology of CPLA. Some authors believe that trauma, cigarette smoking, or the pressure of the cigarette against the lip may contribute to the decrease in the thickness and composition of the connective tissue that supports the arteries, leading to arterial dilation [6, 13]. This dilation can occur particularly in cases of damage to the lower lip; however, a history of trauma or smoking is not present in some cases [14]. Other authors claim that solar damage to the labial mucosa contributes to mucosal degeneration [6, 12]; this can be true for injuries that occur on the lip but does not apply to the injuries to the palate [9] or oral vestibule. The theory of aging states that senile changes, which cause loss of tone of the connective tissue, may also contribute to the formation of a CPLA [2, 6]; however, some cases have been reported in young adults [2]. The most plausible explanation seems to be that of Kocyigit et al. [2], who believe that this anomaly results from a congenital malformation of the blood vessel, which tends to be overlooked for many years until it becomes prominent and pulsating due to senile atrophy [2].

The treatment of choice is surgical removal of the surface vessel, primarily in patients at risk of mucosal biting, those prone to falls from their own height (e.g., alcohol users), and those uncomfortable with the pulsation. Lovas et al. [11] reported that, most of the time, CPLA is a clinical finding and consequently does not require treatment; however, all reported cases associated with ulcers have been treated surgically.

Lovas et al. [11] reviewed the anatomical pathology files from five universities involving 210 cases of CPLA and reported the following primary clinical findings: the presence of pulsating submucosal lesions that affect adults, with no gender preference, and are not associated with lip ulcers or squamous cell carcinoma. CPLA has been described as a common vascular disorder. Prior to this study, only 16 cases of CPLA were described in international journals, indicating that this condition is relatively recent and rarely diagnosed by clinicians and pathologists, but its correct diagnosis is essential for providing proper treatment.
